# Identification of Prognostic Dosage-Sensitive Genes in Colorectal Cancer Based on Multi-Omics

**DOI:** 10.3389/fgene.2019.01310

**Published:** 2020-01-09

**Authors:** Zhiqiang Chang, Xiuxiu Miao, Wenyuan Zhao

**Affiliations:** College of Bioinformatics Science and Technology, Harbin Medical University, Harbin, China

**Keywords:** colorectal cancer, somatic copy number alteration, survival analysis, gene dosage effect, differential co-expression

## Abstract

Several studies have already identified the prognostic markers in colorectal cancer (CRC) based on somatic copy number alteration (SCNA). However, very little information is available regarding their value as a prognostic marker. Gene dosage effect is one important mechanism of copy number and dosage-sensitive genes are more likely to behave like driver genes. In this work, we propose a new pipeline to identify the dosage-sensitive prognostic genes in CRC. The RNAseq data, the somatic copy number of CRC from TCGA were assayed to screen out the SCNAs. Wilcoxon rank-sum test was used to identify the differentially expressed genes in alteration samples with |SCNA| > 0.3. Cox-regression was used to find the candidate prognostic genes. An iterative algorithm was built to identify the stable prognostic genes. Finally, the Pearson correlation coefficient was calculated between gene expression and SCNA as the dosage effect score. The cell line data from CCLE was used to test the consistency of the dosage effect. The differential co-expression network was built to discover their function in CRC. A total of six amplified genes (NDUFB4, WDR5B, IQCB1, KPNA1, GTF2E1, and SEC22A) were found to be associated with poor prognosis. They demonstrate a stable prognostic classification in more than 50% threshold of SCNA. The average dosage effect score was 0.5918 ± 0.066, 0.5978 ± 0.082 in TCGA and CCLE, respectively. They also show great stability in different data sets. In the differential co-expression network, these six genes have the top degree and are connected to the driver and tumor suppressor genes. Function enrichment analysis revealed that gene NDUFB4 and GTF2E1 affect cancer-related functions such as transmembrane transport and transformation factors. In conclusion, the pipeline for identifying the prognostic dosage-sensitive genes in CRC was proved to be stable and reliable.

## Introduction

Colorectal cancer (CRC), is the 3^rd^ leading cause of cancer-associated deaths in the world (Siegel et al., 2019). Studies have shown that somatic copy number alteration (SCNA) is one of the most common and important structural mutations in CRC (Li et al., 2017; Oliveira et al., 2018). SCNA genes are usually considered as the driver gene for cancer development and an important factor for the progression of CRC (Wang et al., 2009; Rosenberg et al., 2018; Lee et al., 2019).

In addition to this few SCNA genes are also being considered as prognostic markers for CRC patients (Roy et al., 2016; Sefrioui et al., 2017). Previous research has shown that a high copy number of mitochondrial DNA can help in identifying the poor prognosis associated with advanced-stage CRC patients (Wang et al., 2016). However, the reason for this specific attribute is still unknown. SCNAs are generated by chromosomal rearrangement. Another important mechanism of SCNA influencing cancer progression is through the gene dosage effect (Harel and Lupski, 2018; Salpietro et al., 2018). For a gene in the region of SCNA, if its expression increases with amplification of the copy number and *vice versa*, this gene would be defined as dosage-sensitive gene. With respect to the unstable and complex nature of expression regulation, the DNA copy number is relatively more stable. Therefore, the copy number of dose-sensitive genes is more likely to be used as a driver gene in cancer. Some of the dosage-sensitive genes (DSGs) such as CD274/PD-L1 gene amplification (Lee et al., 2018b), fibroblast growth factor 1 amplification (Bae et al., 2019), RING-Finger Protein 6 amplification (Steinman et al., 1979), have been shown to be associated with poor prognosis, suggesting DSGs can also be considered as prognostic markers.

The amount of SCNA can be considered as one important indicator of cancer progression. Cancerous tissue may contain both tumor and non-tumor cells, and the copy number of DNA in all cells can be measured during detection. The copy number value obtained from the whole tissue sample with respect to the control sequence reflects the frequency of copy number alteration in the whole sample. This value is often in parts. However, identifying a threshold value of SCNA to be considered as pathogenic or mutant needs a thorough investigation. Jianxin Shi et al. identified significant CNVs using the FASST2 algorithm and selected the number of probes per fragment >5 and log2ratio greater than 0.3 as amplification gene (Shi et al., 2016). Villela et al. also used 0.3 as the SCNA threshold (Kostolansky et al., 1986; Villela et al., 2018). In addition, the copy number amplification or deletion of 0.5 (*i.e.* half amplification or deletion) is pathogenic (Birchler et al., 2001; Birchler and Veitia, 2012). These results suggest that different threshold values should be used as a measure of SCNA.

Due to the importance of DSGs and the fact that SCNA could be a prognostic marker of CRC, we hypothesize that the dosage-sensitive prognostic genes should also affect CRC progression. TCGA is a milestone project of cancer genome covering CNV, RNA-seq data, and patient-specific data of CRC. It can provide a possibility for relatively large-scale excavation of prognostic genes of CRC. In this paper, we have established a pipeline for screening prognosis sensitive genes in CRC, organically identified stable prognostic markers with dosage sensitivity of copy number in CRC, and verified their dosage sensitivity by cell line data. This analysis can help to further enhance our understanding of the value of the prognostic gene of SCNA and can lay a foundation for further analysis.

## Materials and Methods

### Datasets and Processing

The data of CNA, RNA-seq data, and clinical data of CRC were downloaded from the TCGA database. By mapping the copy number probe across the reference genome of hg38, the SCNA at gene level was calculated using Gistic2 software (Mermel et al., 2011). The value of SCNA represents the portability of copy number alteration and the *q*-value for the genes in aberrant regions. The *q*-value > 0.1 and *q*-value < −0.1 were considered as copy number amplified and deleted, respectively. For each gene, the samples with SCNA value > = x (x represents the threshold of SCNA with a value >0) were identified as copy number amplification samples (CNAS), the samples with SCNA < = −x were identified as copy number deleted samples (CNDS), and the samples with | SCNA | < x were identified as copy number non-altered samples (CNNS). The location information of chromosomes was obtained from the HGNC database (Braschi et al., 2019). RNAseq FPKM data was downloaded from University of California Santa Cruz (UCSC, http://genome.ucsc.edu/), and more than 80% of genes with 0 value were filtered out. The test data-set was collected from the Cancer Cell Line Encyclopedia (CCLE; http://www.broadinstitute.org/ccle/home).

### Filtering of Prognosis-Sensitive SCNA Genes

PSGs of SCNA were screened in five steps as described below:

Step 1: Set x (x > 0) as the threshold for SCNA, then the samples of CRC were classified into three groups, somatic copy number amplification samples (CNAS), somatic copy number deletions samples (CNDS), and somatic copy number non-alteration samples (CNNS). The number of CNAS or CNDS was more than or equal to 10. Wilcoxon rank-sum test was performed to identify differentially expressed genes between CNAS and CNNS and between CNDS and CNNS. The *p*-value was corrected by the Benjamini-Hochberg method. As there were very small differences in gene expression between SCNA and CNNS samples their false discovery rate (FDR) < 0.1 and *p* < 0.01, fold change >1.2 were considered as differential expression.
Step 2: In order to further screen the candidate genes on the basis of Step 1. We identified genes with expression up-regulation (*p*-value < 0.01 and FC > 1.2) and copy number amplification (SCNA > x) in CNAS, and the genes with expression down-regulation (*p*-value < 0.01 and FC < 1/1.2) and copy number deletion (SCNA < −x) in CNDS as candidates for the dosage-sensitive gene.
Step 3: The data of SCNA and survival time of all the samples for each abnormal candidate gene was analyzed by Cox regression and the genes with *p*-value < 0.05 were identified as candidate PSG.
Step 4: In order to further screen stable SCNA-PSGs, the SCNA threshold x was raised from 0.1 to 0.5 with 0.02 steps, and the cancer samples were divided into CNAS, CNDS, and CNNS. For each threshold of SCNA, the log-Rank test was used to assess the significance of overall survival times in CNAS vs. CNNS and CNDS vs. CNNS groups. The abnormal driver genes with the number more than 50% number of the thresholds were selected as a stable PSG.
Step 5: In order to further screen dosage-sensitive genes from stable PSGs in different SCNA threshold, the prognostic sensitive abnormal genes of DSGs were selected. Linear regression was applied to assess the dosage-sensitivity. The R-value represents the dosage-effect score. The genes with the *p*-value < 0.05 and *R* > = 0.3 were considered as prognostic dosage-sensitive genes (PDSGs).

### Verification of DSGs in Cell Lines

In order to verify the stability of the dosage-sensitivity of PDSGs, the correlation coefficients between gene expression and copy number alteration were calculated with the RNA-seq of CRC and CNA at gene level downloaded from the CCLE database. These values were compared with the findings obtained from TCGA.

### Building the Differential Co-Expression Network

In order to further identify the genes affected by PDSGs, Pearson correlation coefficients of these six PDSGs and other genes was calculated as co-expression values in CNAS or CNDS, CNNS. Gene pairs with correlation coefficients higher than 0.5 in one group and less than 0.1 in another group were screened as differentially co-expressing gene pairs. Network visualization tools were executed using Cytoscape (Shannon et al., 2003).

### Analysis

All the analysis was performed in the R computing environment. Survival curves were estimated using the Kaplan-Meier method. Gene function enrichment was performed using the Cluster Profiler package (Yu et al., 2012).

## Results

### PDSGs in CRC

A total of 448 CRC samples with SCNA and RNA-seq data were downloaded from The Cancer Genome Atlas (TCGA). The samples were screened for survival information. There were 22,752 genes, of these 17,442 were protein-coding and 14,688 were differentially expressed.

**Figure 1:**
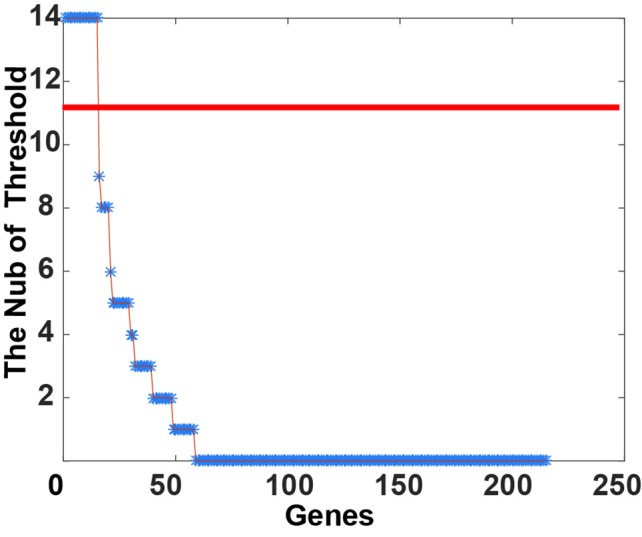
Classification stability of gene prognosis. For each threshold of somatic copy number alteration (SCNA) (from 0.1 to 0.5, at 0.02 step), the *p*-value was calculated by the log-rank test in corresponding alteration and CNNS samples. The Number of Threshold will increase if the *p*-value < 0.05.

After applying FDR < 0.1 and FC > 1.2, 6,814 genes had up-regulated expression in CNAS. Twenty-five genes had a down-regulated expression in CNDS. Cox regression analysis was applied to calculate the correlation between SCNA and survival time. A total of 215 prognosis-sensitive genes (PSGs) significantly related to SCNA were obtained, of these 214 were amplified and one was deleted. Next, the 21 SCNA threshold value was raised from 0.1 to 0.5 at a step of 0.02. For each threshold, the samples were classified into CNNS, CNAS, CNDS group and logRank test between CNNS and CNAS, CNDS and CNNS was performed. As shown in **Figure 1**, 73.02% of genes didn’t show any significant classification with any threshold. A total of 15 genes showed stable prognosis classification of patients in more than 10 threshold values, suggesting these 15 genes can be considered as stable markers for prognosis classification in CRC.

**Figure 2:**
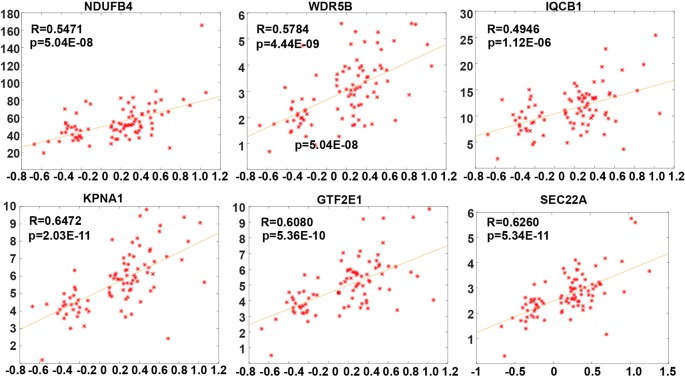
The dosage sensitivity of six prognostic dosage-sensitive genes (PDSGs). The X-axis represents the somatic copy number alteration (SCNA) value and Y-axis represents the FPKM of genes.

After further screening stable PSGs which are highly affected by copy number dosage effect, the Pearson correlation coefficient between copy number and corresponding expression value (FPKM) of these 15 genes was calculated. Finally, six genes (NDUFB4, WDR5B, IQCB1, KPNA1, and SEC22A) which are stable PSGs (**Figure 2**) were identified. The average dosage effect score was 0.5918 and the variance was 0.066.

**Figure 3:**
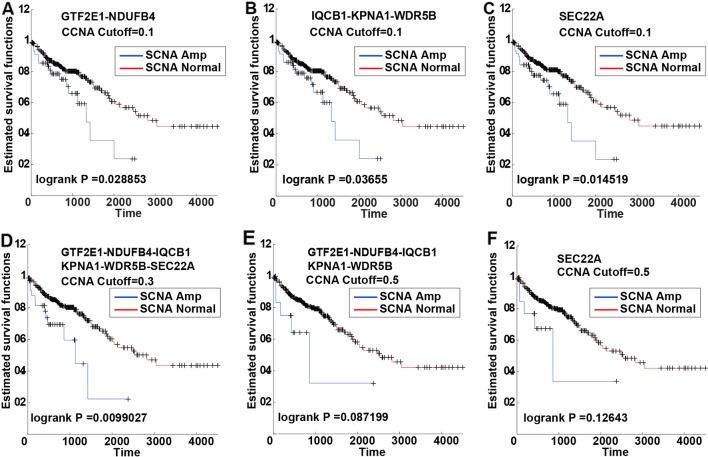
The Kaplan-Meier curves of six PSDGs for samples in CNAS and CNNS. **(A–C)** with the somatic copy number alteration (SCNA) threshold 0.1, gene GTF2E1 and NDUFB4 had similar prognostic classification efficacy. **(D)** with the SCNA threshold 0.3, all six PSDGs have similar efficacy. **(E, F)** with the SCNA threshold 0.5, although the *p*-value was > 0.05, the two survival curves still separated from each other.

Kaplan-Meier survival curve analysis revealed six (6) PDSGs with similar results in a different threshold of SCNA. In the 0.1 SCNA threshold value, genes GTF2E1, NDUFB4, IQCB1, KPNA 1 and WDR5B had a significant classification effect (**Figures 3A–C**). At the 0.3 threshold value of SCNA, all six genes had a similar and significant classification effect (**Figure 3D**). At the 0.5 threshold value, five genes (GTF2E1, NDUFB4, IQCB1, KPNA1, WDR5B) had similar classification effect (**Figures 3E, F**). Although the statistical significance of the two classifications (*p*-value = 0.087199 and *p*-value = 0.12643) in 0.5 SCNA threshold was not significant, their classification curves were distinctly separated. The non-significance can be primarily attributed to the very small number of samples with SCNA threshold >0.5.

### Testing Dosage Effect of PDSGs in CCLE

**Figure 4:**
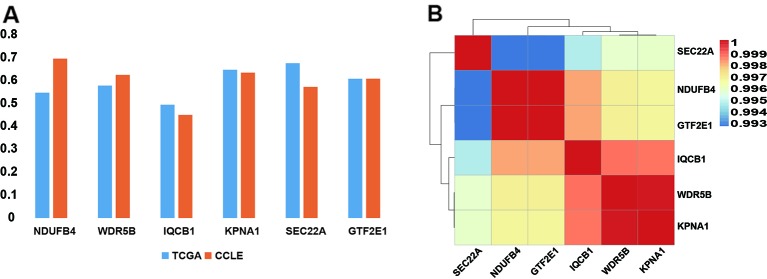
The dosage-sensitive and the correlation scores of somatic copy number alteration (SCNA) of prognostic dosage-sensitive genes (PDSGs). **(A)** The correlation coefficient of SCNA and gene expression. Both results suggest strong concordance. **(B)** The heatmap of the SCNA of PDSG. All these six PDSGs show high co-alteration in colorectal cancer (CRC).

In order to verify if the copy number of six PDSGs is dosage-sensitive in the data from cell lines with 53 cell line samples, the dosage effect score of these six PDSGs in CRC from CCLE was calculated. An average score of 0.5978 and variance was 0.082 consistent with the result from TCGA was obtained (**Figure 4A**). The Pearson correlation coefficient was 1, suggesting that the gene dosage effect is stable in CRC different data.

### Six PDSGs Are Co-Alteration in CRC

Further to test similarity between survival curves of these six PDSGs, we mapped them to chromosomes and found that they all are located on 3q13.33–3q21.1. By computing the correlation coefficients between the copy number of two pairs of genes an average value of 0.9967 (**Figure 4B**) was observed. This indicates that these six PDSGs are highly consistent with each other during alteration.

Research have shown that heterogeneity of copy number alterations exists in ongoing unstable chromosome in COAD (Bolhaqueiro et al., 2019). There are some chromosomes fragile sites in genome, the genes in fragile sites may break when they fell external pressure. In order to determine the presence of breakpoints in the region near to 6PDSGs, they were mapped on the database of human chromosomes fragile sites (HumCFS, http://webs.iiitd.edu.in/raghava/humcfs/). As a result, FRA3D (3q25.32) and FRA3C were found to be near to six PDSGs. Correlation analysis of SCNA in six PDSGs and the genes in FRA3D and FRA3C was performed. Gene RSRC1 (*R* = 0.82), MLF1(*R* = 0.82) in FRA4D, and LPP (*R* = 0.80) in FRA3C had lowest relationship with PDSGs. Thus we infer that the breakpoints in fragile site may explain the reason for the nearby region and a similar SCNA value.

### Building and Analysis of Differential Co-Expression Network With PDSGs

**Figure 5:**
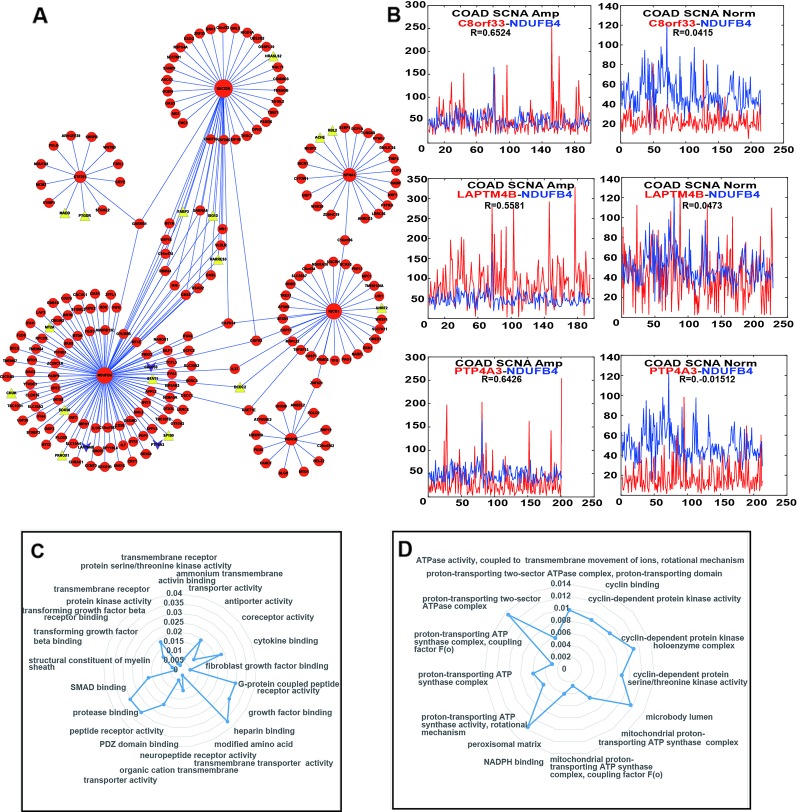
Differential co-expression network and function of enrichment of prognostic dosage-sensitive genes (PDSGs). **(A)** Differential co-expression networks, Triangle represent tumor suppressor genes, lower triangular represent driver gene. Six PDSGs (NDUFB4, WDR5B, IQCB1, KPNA1, GTF2E1, and SEC22A) have the top degree. The edge represents co-expression of the adjacent genes above 0.5 in one group and below 0.1 in another group. **(B)**. The co-expression curve of gene **(C)** Normal and abstained function of gene NDUFB4 using Cluster Profiler R package **(D)** Normal and abstained function of gene GTF2E1 using Cluster Profiler R package.

In order to further explore if these six PDSGs can also affect the expression of other genes in CRC, we screened genes with (*R*) > 0.5 and (*R*) < 0.1 in a different class of samples by calculating the differences of gene co-expression between CNAS and CNNS. A total of 234 co-expressed gene pairs were observed and 215 genes (**Figure 5A**) involved in differential co-expression networks were identified. The whole network constitutes a component suggesting that CRC is a disease involving multiple genes. Among these 194 gene pairs were co-expressed in alteration samples (*R* > 0.5), but not co-expressed in non-alteration samples (*R* < 0.1), while the other 40 pairs behaved in a reverse manner. In the network, gene NDUFB4, SEC22A had the highest degree (109 and 45 respectively) consisting of 15 co-linked genes. The genes CAPN14 and CMPK2 were affected by three PDSGs (NDUFB4, SEC22A, and IQCB1). This suggests that PDSGs are closely linked and interact with each other.

Each PDSG in the network was related to at least 13 genes and 22 genes were associated with more than one PDSG. We also found that several PDSGs-associated genes were also COAD-related. The co-expression of GTF2E1-WNT8B was activated in CNAS(*R* = 0.59). WNT8B one member of the WNT signal was differentially expressed in COAD (Neumann et al., 2014). In addition to this, after mapping the PDSG-related genes to the driver gene list from DriverDB (Liu et al., 2019), three genes (C8orf33, LAPTM4B, PTP4A3) were found (**Figure 5B**), and they all were co-expressed with gene NDUFB4 in CNAS but not in CNNS. Mapping of PDSG-related genes on the tumor suppressor database (TSGene, http://bioinfo.mc.vanderbilt.edu/TSGene/) revealed 16 TSGs (**Figure 5A**, Triangle). Among these, gene DCDC2, ISG15, RARRES3 can affect more than one PDSG. Gene RARRES3 has been shown to be mutated, differentially expressed and also inhibits metastasis in COAD (Lee et al., 2018a). ISG15 is shown to have significant differential expression in COAD (Yu et al., 2019; Zamanian-Azodi and Rezaei-Tavirani, 2019).

Further to explore the possible functions of these six PDSGs, linked genes were extracted and gene ontology function enrichment analysis was performed. Genes linked to gene NDUFB4 (**Figure 5C**) were mainly enriched in functions such as “transmembrane receptor,” “transmembrane transport,” “peptide receptor,” “G protein-coupled receptor,” “transforming growth factor.” Genes linked to gene GTF2E1 were enriched (**Figure 5D**) in functions such as “cyclin-dependent protease,” “ATP synthase transport proton-related functions.” Previous studies have shown that transforming growth factor can also promote tumorigenesis (De Miranda et al., 2015; Yu et al., 2018; Kim et al., 2019). G-protein-coupled receptors (GPCRs) are a member of the largest cell surface molecule family involved in signal transduction and are considered as the key molecule in the growth and metastasis of tumors (Wielenga et al., 2015; Insel et al., 2018). Malignant cells often hijack the normal physiological functions of GPCRs to survive, proliferate independently, escape the epidemic system, increase blood supply, invading the surrounding tissues and spread to other organs.

## Discussion

In this manuscript, a series of screening methods were established to identify PDSGs in CRC. A total of six PDSGs identified in the present study not only have the robustness to different SCNA threshold in prognostic classification but also have the same dosage effect in CRC cell lines. This indicates that our screening pipeline is suitable, reasonable, and effective. The amplification of the copy number of these six PDSGs can lead to poor prognosis, indicating that the SCNA of genes could serve as an important prognostic marker in CRC.

In addition to the stable results, these PDSGs have been shown to be associated with CRC. Gene NDUFB4 encodes a non-catalytic subunit of the NADH. The NADH dehydrogenase complex I is overexpressed in incipient metastatic murine CRC cells (Marquez et al., 2019). Mutations in mitochondrial NADH dehydrogenase subunit 1 (mtND1) gene were found in CRC (Yusnita et al., 2010). WDR5B encodes a protein containing several WD40 repeats, and it is reported as an important target of miR-31. The knockout of microRNA-31 promotes the development of colitis-associated cancer (Liu et al., 2017). The protein encoded by gene SEC22A belongs to the member of the SEC22 family of vesicle trafficking proteins. It has a similarity to rat SEC22 and may act in the early stages of the secretory pathway, which is related to CRC (Jilling and Kirk, 1996; Baron et al., 2010).

Compared with the gene expression the DNA copy number often occurs in arm-level, *i.e.* the same segment tends to have the same copy number alteration (Roy et al., 2016; Xu et al., 2018). The results of this study not only support this opinion but also suggest that even in the same fragment the correlation between different samples is not always 1. There are some differences indicating that somatic alterations have some heterogeneity, and demonstrates the diversity of alteration in CRC. In addition, although chromosomes play a role through the dosage effect to some extent they may be affected by the regulation of gene expression. Six of the 15 genes obtained in this paper have a strong dosage effect suggesting that not all gene copy number amplification will lead to up-regulation of expression. The contribution is a combination of copy number and dosage effect. In future, if targeted drugs or therapies can be developed to reduce the copy number of these six PDSGs, patients with amplified copies of these six genes may receive a precise treatment. This is also an important starting point and foothold of this topic.

The ratio of amplified and non-amplified samples of CPCDGs gene is 1:11, which indicates that these prognostic markers are valuable only for patients with high SCNA. Therefore, SCNA can be an important part of precise medical treatment. Due to computational limitations, the minimum alteration sample selected in this paper is 10, which may reduce the excavation of alteration genes to a certain extent. However, it is believed that in the future, with the increase of the sample size, the increase of different DNA copy number alteration types in CRC will lead to the identification of much clinically relevant SCNA genes.

In summary, the findings of the present study suggest that PDSGs obtained from the analysis of CRC have good application value and can provide an important reference for the precise treatment of CRC.

## Data Availability Statement

All datasets generated for this study are included in the article/supplementary material.

## Author Contributions

WZ designed and supervised the study and was a major contributor in editing the manuscript. ZC analyzed and interpreted the data and was a major contributor in writing the manuscript. XM performed analysis and contributed to the manuscript. All authors read and approved the final manuscript.

## Funding

This research was funded by the Fundamental Research Funds for the Provincial Universities (31041180039).

## Conflict of Interest

The authors declare that the research was conducted in the absence of any commercial or financial relationships that could be construed as a potential conflict of interest.
